# Chronic Physiological Dysregulation and Changes in Depressive Symptoms: Testing Sex and Race as Vulnerability Factors

**DOI:** 10.1007/s40615-024-02189-5

**Published:** 2024-10-10

**Authors:** Stacey N. Doan, Alexandra S. Aringer, Jessica M. Vicman, Thomas Fuller-Rowell

**Affiliations:** 1 Department of Psychological Science, Claremont McKenna College, 850 Columbia Avenue, Claremont, CA 91711 USA; 2 Department of Human Development and Family Studies, Auburn University, Auburn, USA

**Keywords:** Depression, Allostatic load, Sex, Race, College students, Stress, Weathering

## Abstract

Depression is a growing public health concern that affects approximately 5% of adults in their lifetime (WHO in Depression, 2021). Understanding the biological correlates of depression is imperative for advancing treatment. Of particular interest is allostatic load, a multisystem indicator of chronic physiological dysregulation (McEwen and Seeman in, Ann N Y Acad Sci, 1999). The current longitudinal study examined the association between allostatic load, depressive symptoms, and the moderating roles of sex and race. Participants consisted of 150 young adults (*M*_age_ = 18.81) who reported their demographics and depressive symptoms at T1 and T2, a year and a half later. Allostatic load was computed using indicators of metabolic, cardiovascular, and neuroendocrine functioning. Allostatic load was found to predict changes in depressive symptoms. Moreover, interaction effects models revealed that the associations between allostatic load and depressive symptoms at follow-up were further influenced by sex, such that the relationship was significant for males, with pronounced effects for Black males in particular. Black males may be particularly vulnerable to the mental health consequences of biological dysregulation.

Approximately 3.8% of the global population is affected by depression [1], with females reporting more depressive symptoms than males [1, 3]. Although the prevalence of depressive symptoms is well cited, there is a growing need to understand individual differences in vulnerability to depression. While depression stems from multiple causes, such as physical illness and genetics [4–6], of particular interest is the role of stress [7, 8]. Stress has been referred to as “the silent killer” [9, 10] and is associated with anxiety, cardiovascular disease, cancer, depression, and more [11]. While stress can be operationalized in multiple ways, the current study focuses on the physiological assessment of stress and its correlation with depressive symptoms.

Allostatic load (AL) is a multisystem indicator of chronic physiological stress that is thought to index “wear and tear in the body” [12, 13]. Under stress, the body mobilizes the responses of multiple systems (e.g., neuroendocrine, cardiovascular) to effectively respond to the stressor, initiating with engagement of one of the body’s primary stress response systems, the hypothalamic-pituitary-adrenal axis, or “the flight-or-fight” system [13, 14]. However, prolonged overactivation or repeated activation of these physiological stress response systems burdens the body and results in detrimental health conditions [2, 15]. Rather than looking at any single indicator (e.g., cortisol), AL is a measure that combines multiple markers of biological dysregulation. AL has also been found to be associated with morbidity and mortality, especially in disadvantaged populations [16]. Specifically, AL is associated with depression in older adult populations [17–19] and, relatedly, burnout [20].

However, despite recent reviews suggesting the immense value of studying AL in youth [21, 22], AL as a predictor of mental health in young adults is not well understood. Some debate whether it is a useful marker in young people who are relatively healthier than older adults. At the same time, there is a growing literature examining AL as an outcome of adversity including poverty [23] and discrimination [24]. The lack of longitudinal studies conducted on AL and mental health also makes it challenging to understand the direction of effects. However, a few studies are suggestive. AL was found to partially mediate the relations between childhood maltreatment and depression in middle-aged adults [25]. AL also mediated the relationships between cognitive dysfunction in adolescents and internalizing symptoms 20 years later [26]. Thus, AL may be predictive of depressive symptoms in young adult populations.

Young adults are particularly vulnerable to depression due to a confluence of developmental, psychosocial, and environmental factors. This period is a time of transition to adulthood with significance life changes, including leaving home, entering college or the work force, increased responsibilities, and complex relationships. These transitions are likely to lead to stress and uncertainty.

## The Role of Race and Sex

Race and sex play many important roles when considering both allostatic load and depression. There is mixed literature on gender differences in the self-report of depression symptoms, with some studies finding no significant differences in young adults (college students) [27] and adults [28]. However, some work on gender differences in depressive symptoms find that females are more likely to experience depression as compared to males; 25% of females in the US are diagnosed with depression at some point during their lifetime compared to 10–12% of males [29–32]. In contrast, other studies have revealed opposing findings: a higher prevalence of depression exists in males children [33], with a reduction during the shift to adolescence. A large-scale meta-analysis [34] found that, consistent across symptom measures and diagnosis, gender differences of depressive symptoms declined in adulthood and later stabilized across the lifespan. Similar research using latent growth curve analysis demonstrated that boys’ depressive symptoms accelerated in late adolescence, while girls’ depressive symptoms accelerated early in adolescence and then plateaued [35]. Additionally, one study focusing on college students identified a more significant number of male participants experiencing depressive symptoms of varying degrees over 4 years compared to their female counterparts [36].

With regard to health differences, on most measures, males are thought to be more vulnerable than females [37]. Compared to females, male college students are at a greater risk of disease, injury, suicide, and death [38]. This difference is thought to be both neurobiologically [39] and socially mediated [37]. For example, some research highlights that males have lower levels of protective HDL cholesterol than females, which may help explain why males are at a higher risk factor for coronary artery disease [40, 41]. Another line of research has identified adherence to traditional masculine roles as a risk factor for suicide, depression, and substance use [42, 43]. With regard to AL, some research suggests that males tend to have higher levels of AL [44]. Based on this work, we would expect the association between AL and depressive symptoms to be stronger for males.

Likewise, race also seems to have a unique relationship with both allostatic load and depression. Specifically, White participants are more likely to experience acute depressive episodes than minority populations, but minority populations are more likely to experience prolonged and chronic depression [45]. Relatedly, other studies have found that Black participants have higher AL and faster accumulation of AL as compared to their White counterparts [12, 46, 47]. Additionally, Black individuals in America are more likely to experience unique stressors, such as racial discrimination, which in Black adolescents was positively associated with AL [24]. While few studies have systematically examined the role of race and sex, Bey and colleagues found that the associations between allostatic load and depression were stronger for White females and Black males compared to White males and Black females [48]. The authors suggest that these findings on the interaction between sex and race may be influenced by unmeasured psychosocial stressors that could uniquely impact each sex-race group.

The weathering hypothesis suggests that for Black Americans, lifelong exposure to stressors such as discrimination or social and economic adversity leads to deterioration of health [49]. Black males may be particularly vulnerable to depressive symptoms. Black males are subjected to an overwhelming amount of racism and daily discrimination, with mounting evidence highlighting health disparities as a result of repeated exposure. They also do not appear to benefit from higher levels of income, with evidence suggesting that Black men have higher levels of AL regardless of income levels as compared to White men in the income bracket [50]. College education was also associated with worst health among Black men [51], suggesting that there may be a cost to striving for success [52].

Given that the current literature on the relationship between depression and allostatic load is limited in young adults, we aim to investigate the associations between AL and depressive symptoms in a sample of college students and the moderating roles of race and sex using a longitudinal design. Based on past research, we hypothesize that there will be a significant relationship between allostatic load at baseline and changes in depression after a year and a half. We also hypothesize that race and sex will moderate the relationship, such that the association will be stronger for males of both races (vs. females of both races) and for Black males and females (vs. White males and females). Further, we hypothesize that sex and race will intersect, with the association being strongest for Black males.

## Methods

### Participants

Participants were students (*N* = 150, 56% female; *M*_age_ = 18.81, *SD*_age_ = 0.96) from a large Midwestern university, recruited as part of a larger study on stress and health in Black and White college students (45.3% Black). All Black students in the first and second year and a stratified random sample of White students were invited to participate. They were mailed a letter and pamphlet to their local and permanent address. Follow up emails were also sent. Participants’ socioeconomic status had a wide range, but reflected the average annual income of the university’s population (Range: $2500–$200,000; *M*_income_ = $100,411, *SD*_income_ = $57,741.21). Of the 150 students who completed the baseline visit, 133 returned for follow-up. Inspection of missing data patterns revealed that 6.67% of participants were missing values for parental income, and 14% of participants were missing values for depression during the follow-up. Participants who were missing any data were more likely to identify as Black and more likely to report lower parental income (*t* = 4.49, *p* < 0.001; *t* =  − 3.70, *p* < 0.001). There was no significant difference in AL among participants who returned for follow-up (*M* = 1.11; *SD* = 1.03) versus those that did not (*M* = 1.11; *SD* = 1.03), *t* = 1.22, *p* = 0.234, *d* = 0.317.

### Procedure

Laboratory visits occurred at an on-campus clinical research unit in the university hospital. Participants were interviewed regarding their medical history and health behaviors before completing self-report measures on a computer. Baseline blood samples were collected by research and nursing staff in addition to body measurements and autonomic nervous system functioning, after at least 45 min of resting. Participants also collected overnight urine samples at home and returned them to the lab using an insulated container and cold packs. After a year and a half, participants were offered the opportunity to complete several follow-up surveys. Participants were compensated $75 for the baseline visit and $15 for the follow-up. This study was performed in line with the principles of the Declaration of Helsinki. The university’s IRB approved all study materials and protocols, and all participants gave consent for the data obtained to be used for research publication.

### Measures

#### Demographics

Participants completed a demographics survey near the end of the first laboratory visit. They reported their age, sex (male vs. female), race, and parental income. Their self-reported race was verified using the university’s records. Parental income was rated on a 28-point scale (1 = “Less than $5000”, 28 = “More than $200,000).

#### Depression

The Beck Depression Inventory (BDI-II; [53]) was used to assess depressive symptoms during baseline and follow-up. The BDI-II is a validated 21-item assessment (e.g., “Changes in Sleep Pattern,” “Worthlessness”) that participants rate on a 4-point scale (e.g., 0 = “I do not feel that I am worthless,” 3 = “I feel utterly worthless”). Ratings are summed such that higher scores indicate a higher level of depressive symptoms. Reliability was great, Cronbach’s alpha = 0.82 at baseline and 0.88 during the follow-up.

#### Allostatic Load

Allostatic load (AL) was used to measure the cumulative impact of chronic stress exposure on the body. AL consisted of 12 measures indicating the activity of the sympathomedullary (SAM) system (12-h urinary epinephrine and norepinephrine), hypothalamic-pituitary-adrenal (HPA) axis (12-h, overnight urinary cortisol), cardiovascular system (heart rate, resting systolic, and diastolic blood pressure: BP), metabolic system (waist-to-height ratio, body mass index and body adiposity index: BMI, BAI) and immune system (tumor necrosis factor, interleukin-6, and C-reactive protein: TRF, IL-6, CRP). Metabisulfite, a preservative, was used to preserve urine samples. Urine volume was recorded, then duplicate 10-mL samples were elicited. Next, the catecholamine aliquots were acidified and deep-frozen at − 80 °C until the assays were finished. Epinephrine and norepinephrine were assessed by HPLC with electrochemical detection. Cortisol was assessed using a radioimmunoassay. BP and heart rate were measured with computerized readings (Dinamap Model Pro 100, Critikon) at 2-min intervals while participants sat watching a calming nature video. The second to seventh readings were averaged and utilized as the index for resting blood pressure. Height, waist, and hip measurements were measured in centimeters with a measuring tape. Waist-to-hip ratio was calculated as waist circumference in centimeters divided by hip circumference in centimeters. BMI was determined through weight in kilograms divided by squared height in meters. BAI was calculated as waist circumference divided by height. Enzyme-linked immunosorbent assay (range of 0.16 to 10.0 pg/ml) assessed IL-6, CRP, and tumor necrosis factor.

Creatine was used as a control for all hormonal variables, and anthropometric variable ratios were determined based on age and sex benchmarks. Following previous literature [12, 54–56], AL was calculated as the number of biomarkers for which participants scored higher than the 75th percentile. Because low and high cortisol levels have been linked with dysregulation, participants who scored below the 25th percentile, in addition to above the 75th percentile, were given a 1. AL scores had a possible range of 0–12.

## Results

The Lavaan package version 0.6–2 [57] in R 4.2.2 [58] was used for analyses, and missing data was addressed using full information maximum likelihood. All variables met the criteria for normality assumptions. Descriptive statistics for all variables of interest, including covariates, are reported in Table 1. Independent samples *t* tests suggested that Black students experienced higher levels of depressive symptoms as compared to Whites during baseline (*M*_Black_ = 8.40, SD_Black_ = 5.31; *M*_White_ = 5.44, SD_White_ = 4.30; *t(128.28)* =  − 3.70, *p* < 0.01) and during the follow-up (*M*_Black_ = 10.15, SD_Black_ = 7.78; *M*_White_ = 6.13, SD_White_ = 5.69; *t*(88.71) =  − 3.22, *p* < 0.01). There were no significant sex differences with regard to depressive symptoms during baseline and the follow-up. However, there was a significant difference between Black and White students’ reported parental income, with Black students reporting lower family incomes on average, *t(123.46)* = 3.19, *p* < 0.01). There were no significant sex or race differences with regard to AL.

**Table 1:** Descriptive and bivariate correlations for all study variables

	Black	White	Female	Male				
Variable	M(SD)	M(SD)	M(SD)	M(SD)	1	2	3	4
1. Allostatic load	1.32 (1.16)	1.02 (.96)	1.29 (1.11)	1.00 (.98)	-			
2. Depression (T1)	8.40 (5.31)	5.44 (4.30)	7.36 (5.47)	6.04 (4.23)	.22**	-		
3. Depression (T2)	10.15 (7.78)	6.13 (5.69)	8.07 (6.77)	7.37 (7.05)	.25**	.40***	-	
4. Age	18.90 (1.01)	18.74 (.91)	18.63 (.72)	19.05 (1.16)	− .16*	.07	.09	-
5. Parental Income	$82,880 ($57,590)	$113,560 ($54,600)	$102,180 ($59,940)	$98,250 ($55,340)	− .05	− .27**	− .33***	− .19*

*p < .05; **p < .01; ***p < .001

Next, we conducted bivariate correlations to explore the relationships among all variables of interest (Table 1). Allostatic load and parental income were both significantly correlated to depression at baseline, *r*(148) = 0.22 *p* < 0.01; *r*(138) =  − 0.27 *p* < 0.01, depression during the follow-up, *r*(129) = 0.25, *p* = 0.003;* r*(123) =  − 0.33;* p* =  < 0.001, and age, *r*(148) =  − 0.16, *p* = 0.05; *r*(138) − 0.19, *p* = 0.02. Depression at baseline was also significantly correlated with depression during the follow-up, *r(129)* = 0.40, *p* < 0.01. Given these relationships, parental income, age, and baseline depression will be included as covariates in all analyses.

To test our first hypothesis that allostatic load would predict changes in depression, a linear regression analysis was run with race, age, sex, parental income, and baseline depression as covariates. Allostatic load, parental income, and depressive symptoms at baseline and follow-up were standardized prior to regression analyses. As predicted, results showed a significant positive association between allostatic load and depressive symptoms during follow-up. Results also revealed a negative association between parental income and depressive symptoms (see Table 2; Model 1).

**Table 2:** Main and interactive effects of AL, sex, and race on changes in depressive symptoms

	Model 1			Model 2		
Variable	*β*	SE	95% CI	*β*	SE	95% CI
Allostatic load	.18*	.08	.02, .33	0.33**	.13	.07, .60
Sex	.02	.16	− .27, .34	− .01	.15	− .31, .28
Race	.14	.16	− .05, .60	.16*	.16	.02, .64
Parental income	− .19*	.08	− .36, − .03	− .17*	.08	− .33, − .01
Age	.05	.09	− .12, .22	.09	.09	− .07, .27
Depression at T1	.29***	.08	.12, .45	.27***	.08	.11, .43
AL × Sex				− .38**	.16	− .81, − .18
AL × Race				.25*	.16	.04, .65

Race (0 = White, 1 = Black), sex (0 = male, 1 = female)

*p < .05; **p < .01; ***p < .001

**Fig. 1:**
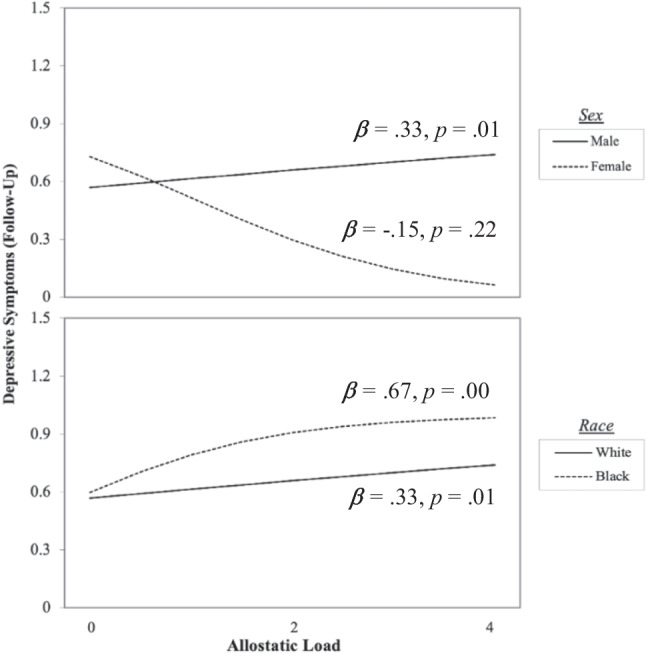
Race and sex moderating the relationship between allostatic load and depressive symptoms at follow-up

We then computed two interaction terms, AL × sex and AL × race, to explore whether race and sex would moderate the relationship between allostatic load and depressive symptoms at T2. The model was significant at *χ*^2^ (8) = 50.792, *p* < 0.001, *R*^2^ = 0.34, indicating an excellent fit of the model to the data, explaining 34% of the variance in depressive symptoms at follow-up. Simple slopes analyses were conducted to examine the relations between AL and depressive symptoms for males versus females and Black as compared to White students. Results as depicted in Fig. 1 demonstrate that AL was associated with increases in depressive symptoms for males (*B* = 0.33, *SE* = 0.13, *p* = 0.01), but not females. Similarly, AL was associated with *greater* increases in depressive symptoms for Black students (*B* = 0.674, *SE* = 0.17, *p* = 0.00) compared to White students (*B* = 0.33, *SE* = 0.13, *p* = 0.01). In sum, findings suggest that depressive symptoms generally increased with higher allostatic load levels in the overall sample. Male participants, particularly Black males, displayed an increase in depressive symptoms in response to increasing allostatic load. However, female participants, regardless of their race, did not display an increase in depressive symptoms with respect to higher allostatic load levels. We further explored relations between the three-way interaction between AL, race, and sex. All previous moderators, interaction terms, and covariates were entered into the regression model. Results demonstrated that the three-way interaction was not significant (*β* =  − 0.25, *p* = 0.19, 95% CI =  − 1.06 to 0.21). This may, however, be due to the substantive larger sample size required to detect three-way interactions.

## Discussion

In the current study, we examine the prospective relations between AL and depressive symptoms in a sample of young adults. We also investigated potential sex and racial differences. We found higher levels of self-reported depressive symptoms in Black as compared to White students. There were no group differences with regard to AL. At both baseline and during follow-up, we did not find any sex differences in depressive symptoms or allostatic load. In line with our first hypothesis, allostatic load was significantly associated with depressive symptoms during the year-and-a-half follow-up, after controlling for race, sex, age, parental income, and prior levels of depression, suggesting that AL may be able to predict the development of depressive symptoms over time. Furthermore, moderation analyses in our models shed light on the influence of sex and race on the relationship between allostatic load and depressive symptoms at follow-up. Particularly, we found significant effects for males and Black young adults, underscoring the critical role examining group variation with regard to understanding the role of physiological stress and its relations to depression outcomes.

Contrary to past findings [47], we did not find racial group differences with regard to levels of AL. This is likely due to the fact that past studies have focused on middle-aged and older adults. Despite the lack of group differences, our data suggests that males and Black students were more vulnerable to the effects of AL over time, providing evidence for the male frailty as documented in physical health domains [37]. With regard to vulnerability to AL, sex differences may also be socially mediated. Hegemonic masculinity and associated standards of men out to be and are judged may make men more likely to engage in unhealthy and risky health behaviors [59], which may exacerbate the relations between AL and depressive symptoms. Moreover, evidence suggests that men are more like to socially withdraw and are more reluctant to seek help [60]. The lack of support for male emotional expression may exacerbate the effects of AL. In fact, some work suggests that stoicism can lead to increases in biological dysregulation [61].

Our data suggesting that Black students are more vulnerable to the effects of AL extend and provide support for the *weathering hypothesis* which posits that the health of Black individuals may deteriorate faster and is more susceptible to insults due to cumulative disadvantage ranging from economic to social [49, 62]. The majority of the work has focused on physical health outcomes and consequent disparities; our data provide evidence for the weathering hypothesis in the psychological health domain as well. Despite no racial group differences in AL levels, AL over time is associated with increases in depressive symptoms for Black youth. Our study has important implications for public health. First, our results add to the growing literature of health disparities among minoritized groups. The significant interactions between race and sex as moderators emphasize the importance of considering intersectionality in research. Our findings underscore the importance of acknowledging interconnected individual experiences and identities (e.g., race, gender, socioeconomic status, disability) and implementing targeted interventions to support the mental health needs faced by different subgroups. Given the increased susceptibility to depressive symptoms among Black males with higher allostatic load, there is a need for targeted mental health screening and support in this population.

Despite the strengths of the current study including the prospective design and objective marker of stress functioning, it also suffers some limitations. Our relatively small sample undermines our ability to detect significant three-way interactions to test intersectional identities. We also use self-reported measures of depressive symptoms. Given sex differences in reporting of symptoms, our results may be strengthened if alternative measures that are not dependent on self-reporting could have been employed. Additionally, since our sample included only Black and White students, differences within other races were not examined. In the future, researchers should examine the differences between other cultural groups to obtain a more accurate representation of the relationship between stress and depressive symptoms. Furthermore, our work is limited by the fact that our sample is a sample of young adults attending college. Indeed, there is a long-standing concern that research using convenience samples of students at 4-year institutions like ours may not be generalizable [63]. Research suggests differences in risk factors and behaviors between both these groups [64], and thus, there is a great need to understand the extent to which the relations between AL and mental health, as well as any potential protective factors, may vary.

Despite these limitations, our data highlights the importance of examining how patterns of differences in susceptibility to health and disease may vary alongside reporting mean levels. In our study, we are unable to tease apart mechanisms for group differences. Future studies may consider biological, psychological, and social mediators of these relationships. In sum, our findings add important nuance to the impact of stress on psychological health and highlights the importance of group differences to vulnerability.

## Data Availability

Deidentified data may be available from request to the first author, depending on human subjects regulations.
